# The impact of bronchial artery embolisation on the quality of life of patients with haemoptysis: a prospective observational study

**DOI:** 10.1007/s00330-020-07533-x

**Published:** 2021-01-06

**Authors:** Naoki Omachi, Hideo Ishikawa, Masahiko Hara, Takashi Nishihara, Yu Yamaguchi, Yumiko Yamamoto, Mihoko Youmoto, Tomoaki Hattori, Kazushi Kitaguchi, Shota Yamamoto, Tomoya Kawaguchi, Masahiro Fukuzawa

**Affiliations:** 1 Hemoptysis and Pulmonary-Circulation Center, Eishinkai Kishiwada Rehabilitation Hospital, Kamimatsu-Chou 2-8-10, Kishiwada, Osaka, 596-0827 Japan; 2 Center for Community-Based Healthcare Research and Education, Shimane University Graduate School of Medicine, Izumo, Japan; 3 Department of Respiratory Medicine, Graduate School of Medicine, Osaka City University, Osaka, Japan

**Keywords:** Bronchial artery, Embolisation, Haemoptysis, Quality of life

## Abstract

**Objectives:**

Patients with haemoptysis often experience daily physical and mental impairment. Bronchial artery embolisation is among the first-line treatment options used worldwide; however, no evidence exists regarding the health-related quality of life (HRQoL) after bronchial artery embolisation. Therefore, this study aimed to evaluate the effects of bronchial artery embolisation on the HRQoL of patients with haemoptysis.

**Methods:**

We prospectively enrolled 61 consecutive patients who visited our hospital from July 2017 to August 2018 and received bronchial artery embolisation for haemoptysis. The primary outcome was the HRQoL evaluated using the Short Form Health Survey, which contains physical and mental components, before and after bronchial artery embolisation. The secondary outcomes were procedural success, complications, and recurrence-free survival rate at 6 months.

**Results:**

The mean age of the patients was 69 years (range, 31–87 years). The procedural success rate was 98%. No major complications occurred. The recurrence-free survival rate estimated using the Kaplan-Meier analysis at 6 months after bronchial artery embolisation was 91.8% (95% confidence interval, 91.1–92.5%). Compared with the pre-treatment scores, the physical and mental scores were significantly improved at 6 months after bronchial artery embolisation (*p* < 0.05).

**Conclusion:**

Bronchial artery embolisation improved the HRQoL of patients with haemoptysis.

**Key Points:**

*• Bronchial artery embolisation improved the HRQoL of patients with haemoptysis*.

*• Vessel dilation on computed tomography and systemic artery-pulmonary artery direct shunting on angiography were the most common abnormalities*.

*• The recurrence-free survival rate estimated using the Kaplan-Meier analysis at 6 months after bronchial artery embolisation was 91.8%*.

## Introduction

Haemoptysis is a potentially life-threatening condition with mortality rates ranging from 7 to 30% for patients with massive haemoptysis [1, 2]. Previously, surgical management was the only available definitive therapy for life-threatening haemoptysis. However, given the higher risks associated with surgery, initial intervention with bronchial artery embolisation (BAE) is currently a preferred alternative and one of the first-line options for haemoptysis [3, 4]. BAE, which uses a bronchial-pulmonary artery shunting mechanism, is an effective therapy for moderate to severe haemoptysis [5, 6]; however, it is associated with several complications, including chest pain, fever, mediastinal haematoma, and aortic dissection. Additionally, post-BAE recurrence rates have been reported to be as high as 10–57.5% [5–8].

Health-related quality of life (HRQoL) is defined as an individual’s overall satisfaction with life and functional status in relation to disease [9]. Haemoptysis often occurs suddenly and without any prodromal symptoms, such as haemo-sputum [5, 10]. Furthermore, patients with haemoptysis experience both acute and chronic decreases in terms of HRQoL [5, 11, 12]. They typically experience anticipatory anxiety related to their fear of death, which is often strengthened by the sudden onset of haemoptysis and restricted physical activity [3, 11]. Patients with severe cases may develop neurosis due to the fear of suffocation and death; however, BAE can potentially improve their HRQoL dramatically [13]. Therefore, it is necessary to determine the measurable post-BAE improvements in HRQoL.

To our knowledge, no study has investigated the effects of BAE on measurable HRQoL. Therefore, we evaluated the effects of BAE on the HRQoL of patients with haemoptysis.

## Materials and methods

### Patient population

We prospectively enrolled all patients who visited the Hemoptysis and Pulmonary-Circulation Center at Eishinkai Kishiwada Rehabilitation Hospital from July 2017 to August 2018, and underwent BAE for haemoptysis. We did not treat haemoptysis caused by lung cancer because previous related studies of BAE were limited by their sample size; moreover, its efficacy and safety remain unclear [14–16]. Our BAE candidates were patients diagnosed with haemoptysis with a volume ≥ 20 mL/day and worsened quality of life. We defined haemoptysis < 39 mL/day, 40–199 mL/day, and ≥ 200 mL/day as mild, moderate, and massive, respectively [4, 5, 12]. This study was approved by the institutional review board of Eishinkai Kishiwada Rehabilitation Hospital (approval date, October 13, 2016; approval number, 2016-001). Written informed consent was obtained through the questionnaires.

### Imaging study interpretation

All the patients underwent pre-BAE CT angiography with contrast medium enhancement to evaluate the baseline disease, bleeding site, and possible haemoptysis-related arteries (HRAs) for procedural planning. CT findings of possible HRAs and bleeding were as follows: vessel dilation, aneurysmal formation, direct vessel shunting, ground-glass attenuation suggesting inhaled blood, tortuosity, hypervascularity, and enhancement of distal part of the HRAs embolised at prior BAE [5, 7, 12]. Three-dimensional images were reconstructed using Ziostation 2 version 2.9.2.2 CT (Canon Aquilion Lightning 80).

### BAE procedure

Trained pulmonologists specialising in the treatment of pulmonary arterio-venous malformations and BAE performed all angiography and embolisation procedures. BAE for aorta-related HRAs was performed using the trans-femoral artery approach, whereas subclavian-related and axillary-related HRAs were performed using the trans-radial or trans-brachial artery approach. The number and approach sites for BAE were determined at the discretion of the attending physician while considering the CT-suggested possible HRAs. All possible CT-defined HRAs were evaluated by selective angiography using a 4-Fr or 5-Fr guiding catheter. In the case of abnormal findings, including systemic artery-pulmonary artery direct shunting and hypervascularity, HRAs were selectively embolised using the 3-Fr microcatheter system with 0.014-in. or 0.016-in. guide wires. Metallic platinum coils (detachable or pushable coils; IDC®, Boston Scientific; Target®, Stryker; Azur®, Terumo Corporation; and C-STOPPER®, PIOLAX Inc.) were used as the embolic agents.

Procedural success was defined as complete HRAs embolisation. We defined the termination of all HRAs embolisation, including its failure, as one series. BAE-naive patients were classified as the initial treatment subgroup. Patients who had previously undergone BAE at least once were classified as the recurrence subgroup. Recurrence events during this study period were classified as haemoptysis ≥ 20 mL/day requiring BAE re-treatment or death due to haemoptysis. If haemoptysis recurred, then repeat BAE was planned.

### Complications

We used intra-procedural and post-procedural observations to determine early BAE complications. Complications were defined using the criteria proposed by the Society of Interventional Radiology Standards of Practice Committee [17].

### Outcome measures

We investigated baseline characteristics, smoking status, comorbidities, baseline pulmonary diseases, modified Medical Research Council scores, hospital stay lengths, procedural success, complications, post-BAE recurrence-free survival rate at 6 months, patient satisfaction with treatment, and HRQoL.

The HRQoL survey was performed as a self-assessment by patients themselves using the SF-8, which is a shortened version of the SF-36 health survey. The SF-8 is the most popular assessment tool, and it is widely used for evaluating HRQoL related to many diseases [18, 19]. Previous studies have confirmed the validity and reliability of the Japanese version of the SF-8 [20, 21]. The raw scores of answers to each SF-8 question were converted to a scale of 0–100, with scores of 0 and 100 indicating the worst and best life quality, respectively. Notably, the reported mean SF-8 score for each summary parameter in the Japanese general population was 50 points. Therefore, all SF-8 assessments were compared with the average score of the general Japanese population [21].

To calculate comparable scores, the SF-8 evaluates eight items regarding health status. These include four physical (physical functioning (PF), role physical (RP), bodily pain (BP), general health (GH)) and four mental (vitality (VT), social functioning (SF), mental health (MH), role emotional (RE)) components. The physical and mental component scores were used to calculate summary measures termed the physical component score (PCS) and mental component score (MCS), respectively [22]. SF-8 scores were obtained using the questionnaire assessment results before treatment and at 1, 3, and 6 months after BAE. Quantitative variables were expressed using mean and standard deviation (SD) values. We calculated the percentage of the change in the score at 6 months compared to the baseline score. All modified Medical Research Council scores were obtained before BAE treatment.

Moreover, we investigated the self-assessed subjective satisfaction with treatment at 6 months after BAE by using the following scale: very satisfied, satisfied, neither satisfied nor dissatisfied, somewhat dissatisfied, or dissatisfied.

### Statistical analysis

The Wilcoxon signed-rank test was used to compare the baseline SF-8 scores with those at 1, 3, and 6 months after BAE. The Mann-Whitney test and chi-squared test were used to compare baseline characteristics of the initial treatment and recurrence groups. The Kaplan-Meier analysis was used to estimate the haemoptysis-free recurrence rate at 6 months after BAE. Statistical significance was defined as *p* < 0.05. All analyses were performed using the statistical package SPSS (SPSS version 22; SPSS).

## Results

### Patient characteristics and clinical outcomes

We excluded eight patients who were lost to follow-up among a total of 69 with haemoptysis who visited the Hemoptysis and Pulmonary-Circulation Center (EHPC) at Eishinkai Kishiwada Rehabilitation Hospital between July 2017 and August 2018. Finally, we enrolled 61 patients with available data who underwent BAE. Table 1 summarises the characteristics of the patients and BAE procedural information. The mean age of patients was 69 years (range, 31–87 years), and 24 (39%) patients were male.

**Table 1:** Patient characteristics and BAE procedural information

Characteristics	No. of patients (*n* = 61)
Age, mean (range)	69 (31–87)
Sex, male/female	24/37
Smoking status, current/ex/never	6/20/35
Body mass index, mean (SD)	20.02 (3.4)
Haemoptysis, mild/moderate/massive	8/14/39
Comorbidity	
Hypertension	14
Malignant disease	7 (hepatocellular carcinoma, 2 cases; oesophageal cancer, 1 case; breast cancer, 1 case; lung cancer, 1 case; bladder cancer, 1 case; prostate cancer, 1 case)
Connective tissue disease	6
COPD	6
Bronchial asthma	6
Previous cerebral infarction	4
Ischaemic heart disease	3
Diabetes mellitus	3
HIV infection	1
Epilepsy	1
Interstitial pneumonia	1
Baseline diseases	
Bronchiectasis/cystic fibrosis	18
NTM	17
Cryptogenic haemoptysis	16
Pulmonary aspergillosis	8
Pulmonary TB sequelae	2
mMRC before BAE (0/1/2/3/4)	12/11/17/12/9
Procedural information for BAE	
Initial/recurrent treatment	37/24
Procedural success rate (%)	198/203 (98%)
Length of hospital stay	5.8 days (95% CI, 4.6–7.1)
Complications	Major complication, none Minor complications: mediastinal haematoma, 1 case; chest pain, 1 case; back pain, 1 case; vomiting, 2 cases; extravasation, 2 cases; vagal reflex, 2 cases
Recurrence-free haemoptysis rate at 6 months	91.8% (95% CI, 91.1–92.5)

*CI*, confidence interval; *COPD*, chronic obstructive pulmonary disease; *HIV*, human immunodeficiency virus; *NTM*, non-tuberculous mycobacteria; *TB*, tuberculosis; *mMRC*, modified Medical Research Council dyspnoea scale; *BAE*, bronchial artery embolisation

There were 14 (20%) and 39 (64%) patients with moderate and massive haemoptysis, respectively. Regarding the BAE series, the initial and recurrence treatment subgroups comprised 37 (61%) and 24 (39%) patients, respectively. The mean hospital length of stay was 5.8 days (95% confidence interval (CI), 4.6–7.1). There were no major complications and nine cases of minor complications: mediastinal haematoma, 1 case; chest pain, 1 case; back pain, 1 case; vomiting, 2 cases; extravasation, 2 cases; and vagal reflex, 2 cases. The recurrence-free survival rate at 6 months was 91.8% (95% CI, 91.1–92.5%). In the recurrence treatment subgroup, baseline diseases were bronchiectasis/cystic fibrosis (10 cases), non-tuberculous mycobacteria (8 cases), pulmonary aspergillosis (3 cases), cryptogenic haemoptysis (2 cases), and pulmonary tuberculous sequelae (1 case). The mean haemoptysis-free interval for this recurrence subgroup was 606 days (95% CI, 415–797 days). Five patients in the recurrent subgroup presented recurrence events during the study period. Three patients underwent repeat BAE uneventfully and haemostasis was achieved. One patient with non-tuberculous mycobacteria (NTM) was treated with repeat BAE; however, haemoptysis continued. Moreover, surgical resection was discouraged due to diffuse pulmonary disease, and the patient died because of respiratory failure under mechanical ventilation support 1 week after the initial BAE treatment. Another patient with haemoptysis caused by aspergillosis died because of respiratory failure secondary to bacterial pneumonia at 3 months after BAE.

**Fig. 1:**
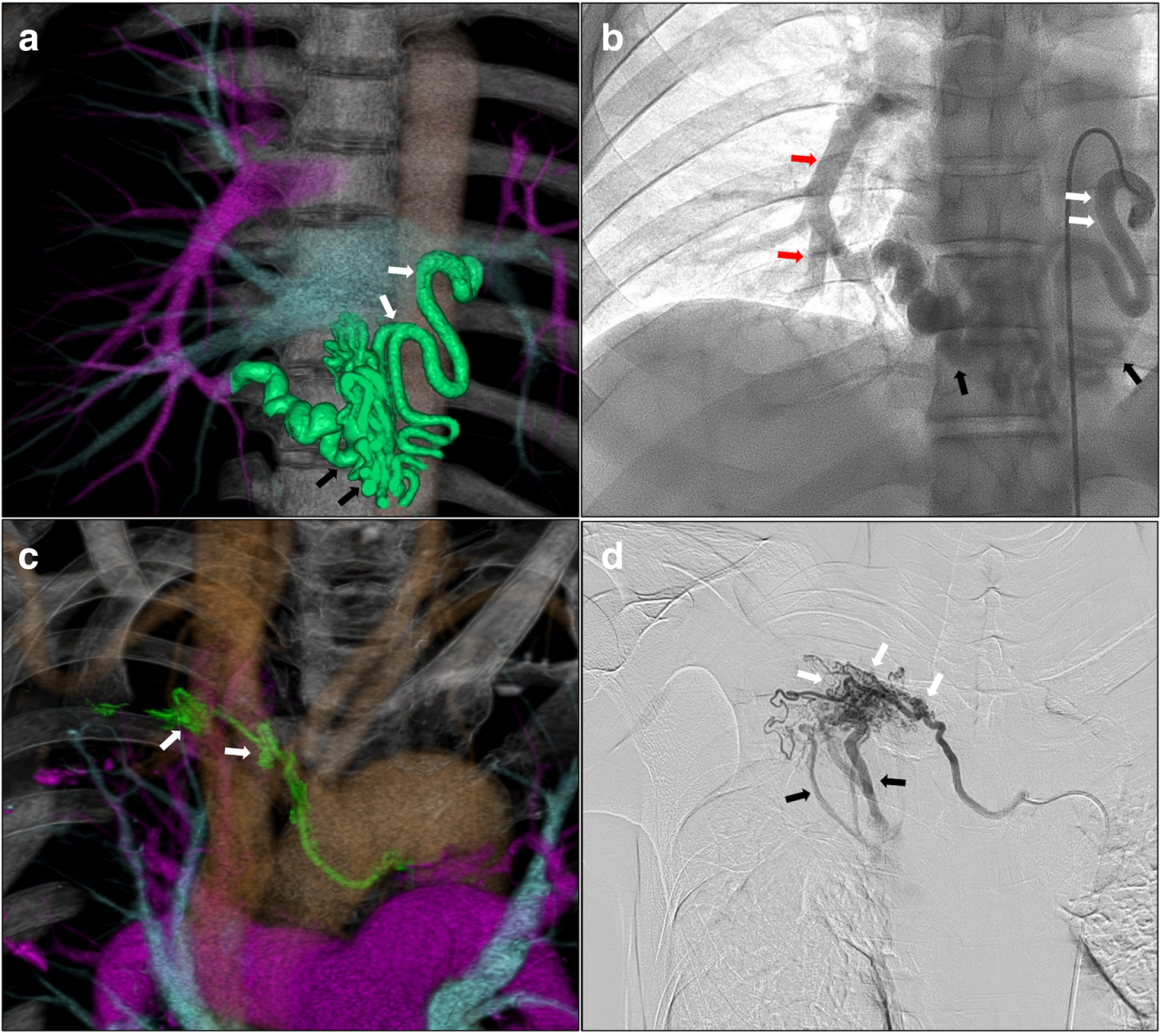
Three-dimensional CT (**a**, **c**) and angiography (**b**, **d**) findings before BAE. **a** Abnormal pulmonary ligament artery with vessel dilation (white arrows) and tortuosity (black arrows). **b** Vessel dilation (white arrows), tortuosity (black arrows), and systemic artery-pulmonary artery direct shunting (red arrows). **c** Abnormal bronchial artery with hypervascularity (white arrows). **d** Hypervascularity (white arrows) and systemic artery-pulmonary artery direct shunting (black arrows)

Table 2 summarises the CT findings, angiographic findings, and HRA information. The most common CT findings were vessel dilation (51%) and tortuosity (26%). The most common angiographic findings were systemic artery-pulmonary artery direct shunting (57%) and vessel dilation (54%). Regarding HRAs, the bronchial artery (44%) was the most common artery, followed by the intercostal artery (18%). Figure 1 shows three-dimensional CT and angiography findings of vessel dilation, tortuosity, hypervascularity, and systemic artery-pulmonary artery direct shunting.

**Table 2:** CT, angiographic findings, and targeted haemoptysis-related arteries

	No. of vessels or cases (%)
CT findings	
Vessel dilation	103 (51)
Tortuosity	53 (26)
Distal enhancement of previous coil embolisation	35 (17)
Hypervascularisation	26 (13)
Ground-glass attenuation (no. of cases)	23 (37)
Aneurysm	0 (0)
Systemic artery-pulmonary artery direct shunt	0 (0)
Angiographic findings	
Systemic artery-pulmonary artery direct shunting	116 (57)
Vessel dilation	110 (54)
Hypervascularisation	75 (37)
Tortuosity	62 (31)
Recanalisation (in recurrence cases)	33 (16)
Collateral (in recurrence cases)	8 (4)
Targeted haemoptysis-related arteries	
Bronchial artery	90 (44)
Intercostal artery	37 (18)
Internal thoracic	21 (10)
Supreme intercostal	13 (6)
Thoracoacromial artery	10 (5)
Lateral thoracic artery	10 (5)
Inferior phrenic artery	9 (4)
Superior thoracic artery	4 (2)
Pulmonary ligament artery	3 (1)
Thoracodorsal artery	3 (1)
Inferior thyroid artery	2 (1)
Dorsal scapular artery	1 (0.5)

### Quality of life analysis

Table 3 shows the individual SF-8 scores before treatment and at 1, 3, and 6 months after BAE. The SF-8 questionnaire was completed by all 61 patients before treatment. Two patients died within 1 month and 3 months, respectively, after BAE; therefore, their subsequent assessments were unavailable. One patient and three patients did not return the questionnaire at 1 month and 3 months, respectively. Among the treated patients, the physical (PCS, PF, RP, GH) and mental (MCS, VT, RE, MH) component scores at 1 month were significantly better than those before treatment (*p* < 0.05). At 3 months, all scores except those for BP and SF were significantly better than those before treatment (*p* < 0.05). At 6 months, the physical (PF, GH) and mental (MCS, VT, RE, MH) component scores were better than those before treatment (*p* < 0.05). Compared with the pre-treatment scores, the scores for PF, GH, and all mental components improved by > 10%, whereas those for the remaining physical components (PCS, RP, and BP) improved at 6 months after treatment (by 9.1%, 7.7%, and 9.3%, respectively).

**Table 3:** SF-8 scores of the entire treated population before treatment and at 1, 3, and 6 months after BAE and comparison of the pre-treatment scores and those at 1, 3, and 6 months after BAE, as well as fractional changes in the score at 6 months after BAE divided by the baseline score and multiplied by 100

	SF-8 mean (SD)				*p*			Change in scores (%) from baseline to 6 months
Total, *n* = 61	Before treatment	1 month after BAE	3 months after BAE	6 months after BAE	Before vs. 1 month	Before vs. 3 months	Before vs. 6 months	
Physical component score	42.81 (9.28)	47.39 (7.45)	47.21 (7.23)	45.29 (8.04)	< 0.001	0.003	0.058	9.5
Physical functioning	40.33 (11.61)	45.76 (9.13)	45.40 (9.05)	43.58 (9.97)	< 0.001	0.008	0.035	15.2
Role physical	41.68 (11.12)	46.31 (8.52)	45.45 (9.45)	42.90 (10.50)	0.002	0.092	0.647	8.1
Bodily pain	51.07 (9.70)	52.96 (7.82)	54.25 (6.81)	53.69 (7.84)	0.171	0.036	0.059	9.3
General health	43.00 (7.31)	50.53 (7.30)	49.53 (6.96)	48.55 (7.74)	< 0.001	< 0.001	< 0.001	15.63
Mental component score	43.18 (9.40)	46.85 (7.51)	46.91 (8.04)	47.37 (8.30)	0.027	0.023	0.002	14.14
Vitality	44.74 (7.32)	49.10 (6.83)	48.86 (6.90)	49.29 (7.32)	0.001	0.007	0.001	12.25
Social functioning	41.81 (11.13)	44.12 (10.26)	45.41 (11.28)	44.69 (10.26)	0.121	0.061	0.059	10.58
Role emotional	41.44 (11.37)	46.09 (7.89)	45.95 (9.01)	45.93 (9.31)	0.001	0.002	0.001	17.89
Mental health	45.43 (9.51)	49.99 (7.29)	49.59 (7.36)	49.06 (8.42)	0.005	0.022	0.013	12.13

*BAE*, bronchial artery embolisation; *SD*, standard deviation

**Fig. 2:**
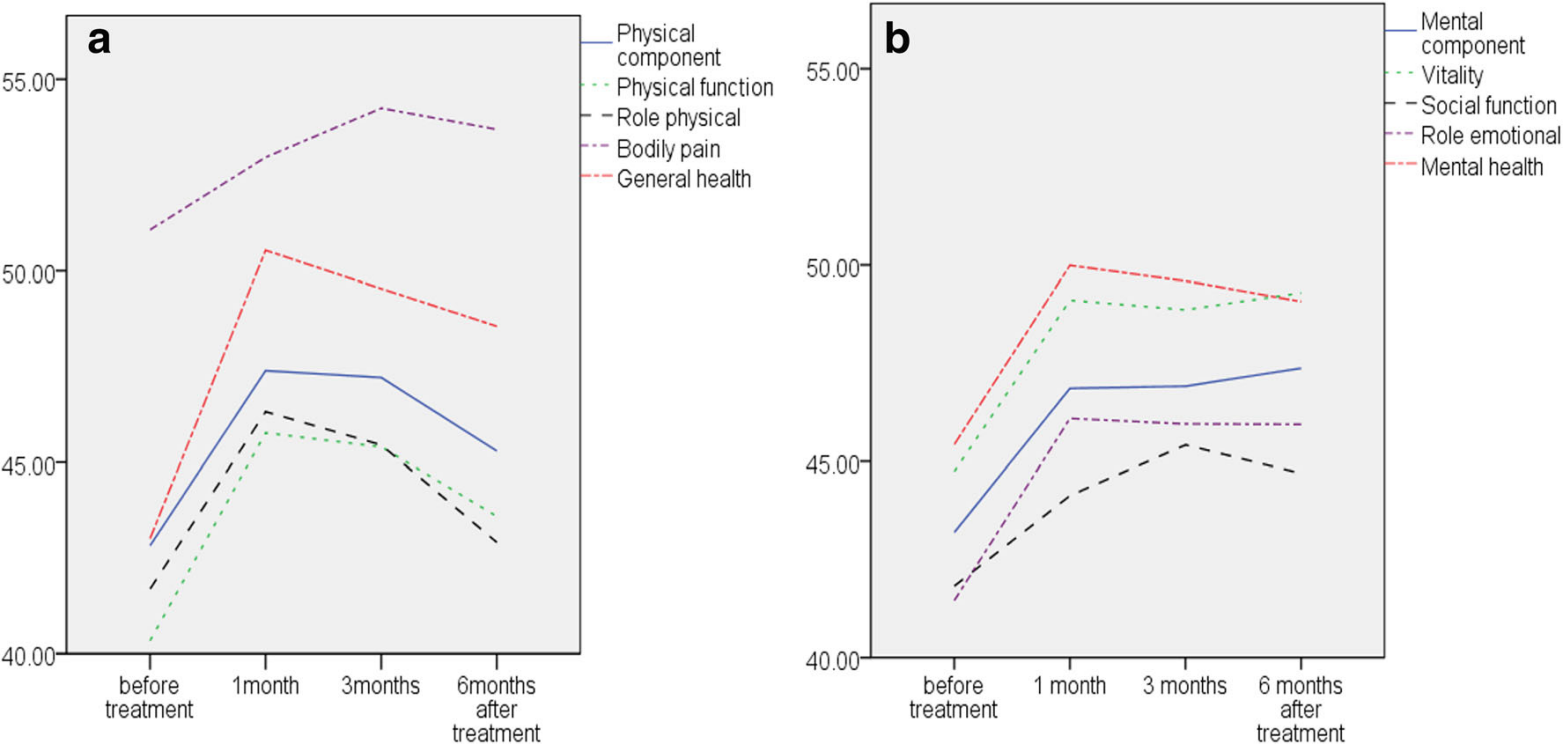
**a** The mean SF-8 scores for the physical component score (PCS) and the PCS before treatment and at 1, 3, and 6 months after treatment. **b** The mean SF-8 scores for the mental component score (MCS) and the MCS before treatment and 1, 3, and 6 months after treatment

Figure 2 visually represents SF-8 score changes from before treatment to 6 months after BAE for the physical and mental components. There was no difference in the baseline characteristics of the initial and recurrence treatment subgroups (Table 4). Compared with the pre-treatment scores, the initial treatment subgroup showed a significant improvement in GH and all mental components at 6 months after treatment (Table 5). Compared with the pre-treatment scores, PF in the recurrence subgroup improved at 6 months (Table 6).

**Table 4:** Baseline characteristics of patients who underwent initial and recurrence treatment

Parameter	Initial treatment, *n* = 37	Recurrence treatment, *n* = 24	*p*
Age, mean (range)	69.5 (46–87)	62.4 (31–82)	0.567
Sex, male/female	13/24	11/13	0.403
Smoking status, current/ex/never smoker	5/12/20	1/8/15	0.478
Body mass index, mean (SD)	20.49 (3.3)	18.28 (4.0)	0.083
Haemoptysis, mild/moderate/severe	7/8/22	1/6/17	0.249
mMRC score, 0/1/2/3/4	9/7/7/9/5	3/4/10/3/4	0.297

*SD*, standard deviation; *mMRC*, modified Medical Research Council dyspnoea scale

**Table 5:** SF-8 scores of the initial treatment subgroup before treatment and at 1, 3, and 6 months after BAE and comparisons of the pre-treatment scores and those at 1, 3, and 6 months after BAE

Total, *N* = 37	SF-8 mean (SD)				*p*		
	Before treatment	1 month after BAE	3 months after BAE	6 months after BAE	Before vs. 1 month after BAE	Before vs. 3 months after BAE	Before vs. 6 months after BAE
Physical component score	44.38 (9.30)	48.51 (8.23)	48.99 (6.08)	46.23 (7.76)	0.002	0.007	0.316
Physical functioning	42.06 (11.89)	47.12 (9.65)	47.60 (7.17)	44.22 (10.44)	0.001	0.011	0.312
Role physical	43.04 (10.62)	47.99 (8.35)	46.90 (8.70)	44.98 (9.88)	0.003	0.059	0.059
Bodily pain	53.19 (8.95)	53.97 (7.44)	55.52 (6.24)	54.96 (7.43)	0.723	0.136	0.315
General health	42.45 (7.17)	51.85 (7.41)	50.13 (6.32)	50.02 (6.19)	< 0.001	< 0.001	< 0.001
Mental component score	43.05 (9.87)	48.33 (7.05)	47.24 (7.70)	49.45 (6.61)	0.007	0.014	< 0.001
Vitality	44.83 (6.74)	49.83 (7.14)	49.84 (6.29)	50.24 (6.69)	0.002	0.001	0.001
Social functioning	42.89 (10.93)	45.81 (10.01)	46.75 (10.59)	47.31 (8.97)	0.097	0.032	0.007
Role emotional	41.63 (11.45)	47.60 (6.77)	46.90 (8.25)	47.20 (9.30)	0.001	0.005	0.004
Mental health	46.08 (9.85)	51.69 (6.28)	50.09 (6.63)	51.45 (6.21)	0.004	0.051	0.008

*BAE*, bronchial artery embolisation; *SD*, standard deviation

**Table 6:** SF-8 scores of the subgroup who underwent recurrence treatment before treatment and at 1, 3, and 6 months after BAE and comparisons of the pre-treatment scores and those at 1, 3, and 6 months after BAE

Total, *n* = 24	SF-8 mean (SD)				*p*		
	Before treatment	1 month after BAE	3 months after BAE	6 months after BAE	Before vs. 1 month after BAE	Before vs. 3 months after BAE	Before vs. 6 months after BAE
Physical component score	40.40 (8.89)	45.50 (5.60)	43.74 (8.16)	43.70 (8.43)	0.040	0.193	0.082
Physical functioning	37.66 (10.87)	43.48 (7.89)	41.10 (10.86)	42.50 (9.25)	0.035	0.498	0.027
Role physical	39.60 (11.77)	43.49 (8.23)	42.61 (10.43)	39.40 (10.80)	0.121	0.759	0.722
Bodily pain	47.79 (10.07)	51.28 (8.30)	51.77 (7.35)	51.57 (8.22)	0.130	0.128	0.087
General health	43.85 (6.72)	48.32 (6.69)	48.36 (8.13)	46.07 (9.45)	0.068	0.288	0.342
Mental component score	43.38 (8.82)	44.36 (7.75)	46.27 (8.84)	43.85 (9.74)	0.970	0.981	0.961
Vitality	44.59 (8.28)	47.87 (6.22)	46.93 (7.75)	47.68 (8.18)	0.190	0.904	0.253
Social functioning	40.16 (11.45)	41.28 (10.28)	42.80 (12.39)	40.27 (10.96)	0.724	0.833	0.858
Role emotional	41.15 (11.48)	43.53 (9.08)	44.09 (10.33)	43.80 (9.13)	0.246	0.203	0.089
Mental health	44.41 (9.07)	47.12 (8.08)	48.62 (8.71)	45.05 (10.15)	0.513	0.372	0.733

*BAE*, bronchial artery embolisation; *SD*, standard deviation

Self-assessed satisfaction grades were obtained from 58 patients: 45% were very satisfied (26/58 patients); 48% were satisfied (28/58 patients); 7% were neither satisfied nor dissatisfied (4/58 patients); and none was somewhat dissatisfied or dissatisfied.

## Discussion

This is the first study to demonstrate the improvement in the HRQoL of patients with haemoptysis treated with BAE. The main findings were that BAE was beneficial for improving the HRQoL of patients with haemoptysis, particularly the mental impairment scores of the initial treatment subgroup, and that the efficacy and safety of BAE could contribute to HRQoL improvement.

We observed significant improvements in the physical (PF, GH) and mental (MCS, VT, RE, MH) component scores at 6 months after BAE compared with the pre-treatment scores of the entire treated population.

Both the SF-8 and SF-36 scores varied according to disease stage, severity, and status, even for patients with similar pulmonary diseases [14, 23]; therefore, the efficacy of the treatment in terms of HRQoL should be evaluated based on the changes and/or relative values compared with the average score of 50 for the general population. However, there is no generally accepted definition of a minimally important change in the quality of life [24]; a 10% difference in the SF-36 summary scores is generally considered a clinically relevant difference [25].

Most mental scores showed a significant improvement at 1 month after treatment, and this improvement was maintained for 6 months. All mental score improvement ratios exceeded 10% within 6 months, with > 90% of the patients reporting satisfaction with the treatment. Given the favourable improvement in mental impairment, we assumed that the patients experienced increased confidence that haemoptysis might not occur suddenly and place them at risk for more severe health outcomes. These results indicate that BAE notably affected the psychological wellness of patients.

Conversely, although most physical component scores improved at 1 month and 3 months, there was a modest decline in scores at 6 months compared with scores at 3 months and in the improvement trend compared with the pre-treatment values. The modest decline in the physical scores at 6 months could be attributed to the following factors:

1. *Haemoptysis recurrence*: Five patients experienced recurrence during this study period. Recurrent events during the study period and the presence of haemo-sputum and mild haemoptysis not requiring repeat BAE might have affected the physical status of the patients.
2. *Worsening of baseline pulmonary diseases*: Baseline pulmonary diseases, including NTM disease and pulmonary aspergillosis, have slowly progressing clinical courses [26, 27] (i.e., the 5-year mortality rates varied between 5 and 42% for NTM [26] and between 38 and 85% for chronic pulmonary aspergillosis [28]).
3. *Comorbidities*: We enrolled elderly individuals with widely varying comorbidities, including stroke, coronary heart disease, malignancy, and connective tissue disease.

Because BAE only controls haemoptysis and not baseline diseases, we considered physical improvements in the HRQoL to be the result of haemostasis induced by BAE at 1 month and 3 months. Moreover, the aforementioned factors are associated, individually or in combination, with a modest subsequent physical decline in the clinical overall outcomes of patients.

Regarding the subgroup analysis, the initial treatment subgroup showed improvements in GH and all mental components at 6 months. These subgroup analyses yielded results similar to those of the entire population; however, in the recurrence subgroup, only PF showed an improvement at 6 months. We previously reported that the procedural success rate of repeat BAE was 97.7% [12]; therefore, repeat haemoptysis can be technically treated using BAE. Nevertheless, all recurrences and death events occurred in the recurrence subgroup. The subgroup analysis showed that BAE resulted in HRQoL improvements in the initial treatment subgroup.

Previous studies reported BAE complications involving vascular injuries, such as vasospasm, aortic dissection, and perforation (0.3–13%) [22, 29–34], and spinal cord ischaemia that led to transient or permanent paraplegia (0.6–4.4%) [8, 35, 36]. There are various types of embolic agents, and complication rates differ across agents. Regarding BAE using a metallic coil, a previous study reported that haemostasis rates were > 90% at 1 year, which are comparable to those for other embolic agents, and that major complication rates were favourable (0–1.4%) [5, 6, 12, 37]. Our findings support the efficacy and safety of BAE using a metallic coil; moreover, the lower invasiveness of BAE was thought to have contributed to the improvement in HRQoL.

This study had several limitations. First, the sample size was small, which limited its statistical power. Second, we did not treat haemoptysis caused by lung cancer; therefore, our findings cannot be applied to patients with lung cancer. We evaluated the HRQoL of patients with haemoptysis, but the study period was short; therefore, we did not evaluate the long-term status of HRQoL improvements after BAE. Because *N*-butyl-2-cyanoacrylate and polyvinyl alcohol are not covered by Japanese public health insurance, we used a metallic coil as the embolic agent. Therefore, it is unclear whether these findings could be applied to other embolic agents. Because two patients died during the study period, their responses could not be obtained. Therefore, the results could have been worse if their responses were included. Moreover, we did not assess the standard of living, educational level, economic status, and family issues, which affect the physical and mental status of patients. Nevertheless, our results indicate that BAE improved the HRQoL of patients with haemoptysis.

## Abbreviations

BAE: Bronchial artery embolisation
BP: Bodily pain
COPD: Chronic obstructive pulmonary disease
GH: General health
HRAs: Haemoptysis-related arteries
HRQoL: Health-related quality of life
MCS: Mental component score
MH: Mental health
NTM: Non-tuberculous mycobacteria
PCS: Physical component score
RE: Role emotional
RP: Role physical
SD: Standard deviation
SF: Social functioning
VT: Vitality
